# Human–Elephant Conflict in Thailand over the Past Decade (2014–2023): Occurrence, Geographical Distribution, and Temporal Trends

**DOI:** 10.3390/ani15091304

**Published:** 2025-04-30

**Authors:** Jarawee Supanta, Chaithep Poolkhet, Marnoch Yindee, Wallaya Manatchaiworakul, Tuempong Wongtawan

**Affiliations:** 1 Akkhraratchakumari Veterinary College, Walailak University, Nakhon Si Thammarat 80160, Thailand; jarawee.su@wu.ac.th (J.S.); poolkhet@yahoo.com (C.P.); drfungy2000@yahoo.com (M.Y.); 2 Centre for One Health Research Center, Walailak University, Nakhon Si Thammarat 80160, Thailand; 3 Clemson Veterinary Diagnostic Centre, Clemson University, 500 Clemson Road, Columbia, SC 29229, USA; wmanatc@clemson.edu

**Keywords:** casualty, crop foraging, coexistence, region, seasonality, wildlife

## Abstract

The endangered Asian elephant, Thailand’s national symbol, is increasingly affected by human–elephant conflict (HEC), now a critical conservation and socio-economic concern. This study examined 341 HEC occurrences in 34 out of 77 Thai provinces between 2014 and 2023, based on data from official and private sources. These events resulted in both human and elephant casualties and property damage. Occurrences peaked in 2018 and 2023, with significant associations found between conflict rates, regions, and periods. The Eastern region and the July-to-October period (the rainy season) emerged as key high-risk areas, likely related to additional factors such as high elephant densities and abundant crop production. Most HECs occurred in agricultural land, where elephants searched for food. The findings suggest that the July-to-October period requires heightened surveillance and additional preventive actions. The Eastern region remains a priority for intervention due to its ongoing vulnerability and the movement of elephants across provincial boundaries. Furthermore, the rise of the Northeastern region as a new hotspot underlines the need for proactive measures to prevent escalation.

## Simple Summary

Thai people and elephants have coexisted for many centuries. However, data analysis from the past decade revealed that human–wild elephant conflict (HEC) has occurred in 44% of provinces, causing significant damage to both humans and elephants. High-risk areas for HEC are characterized by large wild elephant populations, insufficient forest cover, and increased crop cultivation that attracts elephants. The cultivation period poses a higher risk of HEC, with most incidents occurring in agricultural areas, where elephants forage for human food. These findings can inform future HEC prevention strategies.

## 1. Introduction

Elephants (*Elephas maximus indicus*) have been an integral part of Thai culture, history, and national identity for centuries, serving as a revered symbol of the nation [1]. In Thailand, elephants are classified into two distinct populations: captive elephants, which have worked alongside humans for centuries and are now primarily used in the tourism industry [2], and wild elephants, which are classified as an endangered species [3]. The latter population is involved in human–elephant conflict (HEC), leading to significant collateral damage, including human and elephant fatalities, injuries, and property destruction [4].

HEC has been a persistent issue in regions where human activities overlap with elephant habitats. This conflict has been reported in 31 countries, with the majority of reports originating from Asia [5]. Thailand is among the leading countries affected by HEC, which has significant impacts on conservation efforts, society, and the national economy [5,6,7]. HEC is suspected to be driven by agricultural expansion and infrastructure development encroaching upon the natural habitats of wild elephants, largely due to increasing human populations [8,9]. Despite being revered as Thailand’s national symbol, the Asian elephant has received relatively limited research attention regarding HEC, both in terms of the number of studies and the breadth of explored dimensions [6,10]. One national report showed 107 occurrences of HEC across Thailand between 2012 and 2018, resulting in 75 human deaths or injuries and 32 elephants killed or injured, and the trend of injuries for both humans and elephants tends to continuously increase in Thailand [11]. HEC is considered to be a multifactorial issue, influenced by environmental, elephant-related, and human-related factors. Environmental factors associated with HEC include habitat loss, the replacement of natural vegetation with economic crops, and climate or seasonal changes. The conversion of forests into agricultural lands and infrastructure development have significantly reduced elephant habitats, forcing elephants to encroach on human-dominated areas [12,13,14]. Certain economic crops, such as durian, cassava, and sugarcane, are particularly attractive to elephants, drawing them into agricultural areas [6]. Additionally, climate change affects water availability and food sources, exacerbating the conflict by altering elephant migration patterns and increasing their reliance on agricultural areas [7,15,16].

Elephant-associated factors may include population growth, foraging behavior, adaptation, and learning ability. A high number of wild elephants can lead to HEC when elephants and humans compete for food, water, and land. The growth of elephant populations is also affected by many demographic factors, such as births, deaths, immigration, and emigration. These are affected by environmental factors like the quality of the habitat and the amount of water available, as well as human disturbances like poaching and land conversion [17]. Elephants might change their foraging behavior seasonally to meet their nutritional needs. During wet seasons, they prefer grasses, which are more abundant and nutritious, while in dry seasons, they shift to browsing woody plants due to the scarcity of grasses [18]. Moreover, elephants exhibit advanced cognitive abilities, including memory and social learning [17]; they learn to navigate human-dominated landscapes, adapting their behavior to access crops or avoid barriers like fences [19].

Human-associated factors include human population growth, economic status, and societal attitudes. Increasing human populations intensify competition for space and resources, leading to habitat encroachment and fragmentation, which exacerbate conflicts [20]. Lower-income individuals often exhibit lower tolerance toward elephants due to the substantial economic losses associated with crop damage and property destruction [21]. Notably, past experiences with crop damage or safety threats influence community attitudes, often reducing tolerance toward elephants [22,23]. In contrast, communities that benefit financially from elephants, such as those engaged in ecotourism, are more likely to support conservation efforts and coexistence [21].

Although many strategies have been used to resolve HEC [24], the occurrence of HEC is still generally increasing; this could reflect the fact that the root causes of this problem remain unclear and that a resolution has not been entirely achieved since the research on HEC in Thailand is still limited [24]. Therefore, the importance of this research lies in its potential to deepen our understanding of the factors driving national HEC and to inform the development of sustainable management practices. The objective of this study was to investigate the contributing factors of HEC in Thailand based on a decade of data (2014–2023). Ultimately, addressing HEC is essential not only for the protection of elephants but also for promoting sustainable coexistence between human populations and wild elephants in Thailand.

## 2. Materials and Methods

### 2.1. Ethics

This study was approved by the Walailak University Research Ethics Committee (WUEC-24-354-01), as all information was obtained from publicly available online sources provided by private and human casualty data from news sources.

### 2.2. Data Collection

HEC occurrences were collected from Thai online news reported from January 2014 to December 2023 from different online sources. Data collection was conducted using the Google search engine in Thai languages with keywords such as “human, death, injury, elephant, and/or conflict” focusing on incidents that occurred between January 2014 and December 2023. The HEC dataset included details such as date, location, source, number of people involved, number of wild elephants involved, and details of collateral damage. HEC data were recorded in Microsoft^®^ Excel^®^ 2016 (Microsoft Corporation, Redmond, WA, USA) and thoroughly reviewed to eliminate duplication. The raw data for HEC incidents are available to download at DOI:10.17632/fgcxk9f5fw.1.

Forest area data were obtained from the website of the Thailand Royal Forest Department (https://data.forest.go.th (accessed on 1 March 2025)), while wild elephant population data were obtained from the Wildlife Conservation Office (https://portal.dnp.go.th (accessed on 1 March 2025)). Rainfall data were obtained from the National Hydroinformatics Data Center (https://www.thaiwater.net (accessed on 1 March 2025)) and the Thai Meteorological Department (https://www.tmd.go.th (accessed on 1 March 2025)). Human population density was obtained from the Department of Public Works and Town and Country Planning (https://www.dpt.go.th (accessed on 1 March 2025)). Other data were obtained from the National Statistical Office (https://www.nso.go.th (accessed on 1 March 2025)). All data were recorded and subjected to preliminary descriptive analysis using Microsoft^®^ Excel^®^ 2016 (Microsoft, Washington, USA).

### 2.3. Data Visualization

Occurrences of HEC were categorized into 6 regions, Eastern, Northern, Northeastern, Southern, Central, and Western—based on Thai government data. A national map of HEC occurrences was created using ArcGIS Pro 3.4 (ESRI, Redlands, CA, USA). The occurrences were also categorized into three periods throughout the year: March to June (considered the summer season in Thailand), July to October (the rainy season), and November to February (the winter season). The occurrences are presented as the raw number of HEC and mean ± standard error mean (SEM) for each area or season. The collateral damage of HEC was categorized into 3 groups: elephant casualties (injury and death), human casualties, and property damage (agricultural areas, vehicles, and buildings). Forest area data are presented in square kilometers (km^2^), along with the percentage of forest cover relative to the total land area. Both human and elephant population densities are expressed as individuals per square kilometer (individuals/km^2^). Human population density was calculated by the government using the following formula: total population divided by total land area. Elephant population density was determined by dividing the wild elephant population by the forest area in 2023.

### 2.4. Statistical Analysis

Statistical analysis was conducted using the R program (version 3.4.0) (R: The R Project for Statistical Computing, 2023). Geographic and seasonal effects on HEC were analyzed by generalized estimating equations (GEEs) to evaluate differences across regions and periods, and further multiple comparisons were conducted using Tukey’s post hoc tests. Furthermore, generalized linear models (GLMs) with a Poisson model were used to analyze the relationship between the independent variables (regions and periods) and the dependent variables (HEC occurrences), as well as to assess interactions between the independent variables and the dependent variables in order to determine potential confounder interactions. Statistical significance was defined as *p* < 0.05 for all tests. Spearman’s rank correlation coefficient was used to test the correlation between annual HEC patterns and wild elephant population, forest area, and rainfall patterns.

## 3. Results

### 3.1. Occurrences of HEC

**Figure 1:**
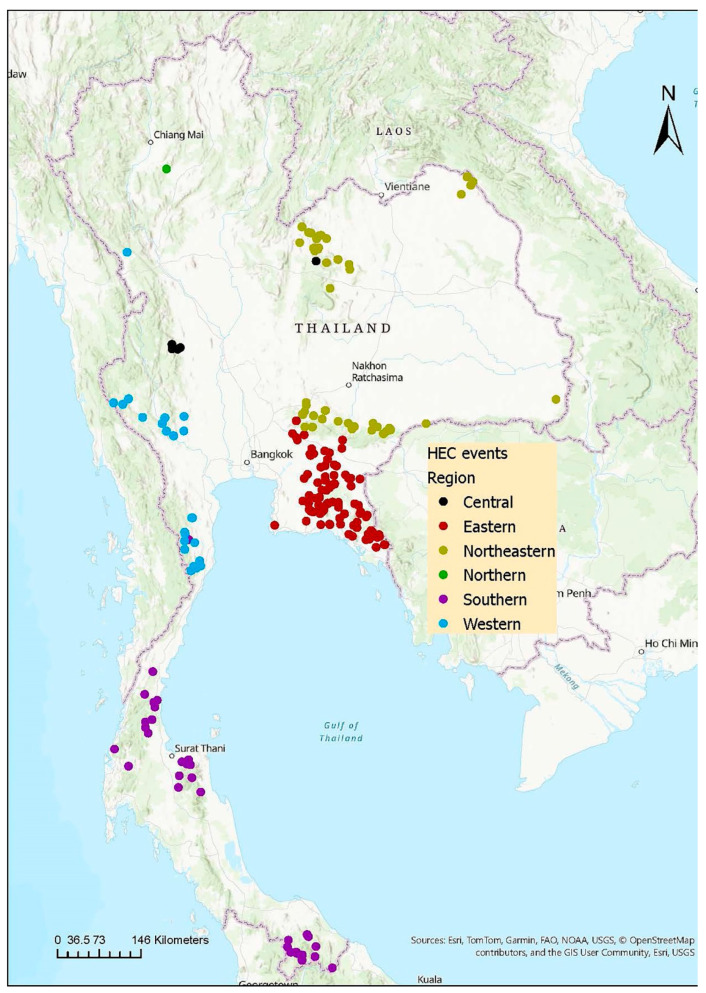
Geographical distribution of occurrences of human–wild elephant conflict in Thailand from January 2014 to December 2023. Each color dot represents one HEC incident.

HEC was reported by 30 news agencies, documenting 341 occurrences of human–wild elephant conflicts across 34 out of 77 provinces in Thailand (44.15% of the country). Each occurrence is detailed in Figure 1.

**Figure 2:**
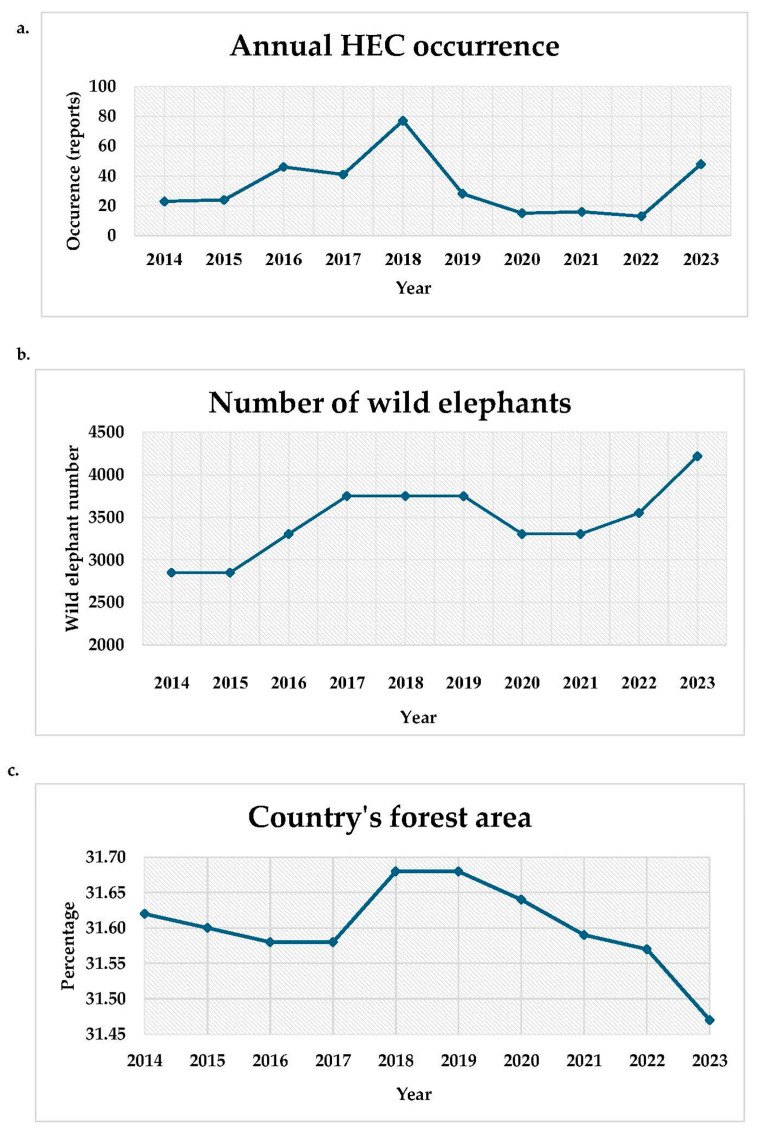
Annual trends in national HEC occurrence (**a**), wild elephant population (**b**), and forest area (**c**) from 2014 to 2023.

The annual total occurrences of HEC in Thailand exhibited a fluctuating trend over the past decade, as shown in Figure 2a. The number of reports increased from 2014 (n = 23) to a peak in 2018 (n = 79), followed by a sharp decline in 2020 (n = 17). The occurrences remained relatively stable until 2022 before rising dramatically in 2023 (n = 48). In 2023, the Northeastern region recorded the highest number of cases, with Loei province reporting more incidents than any other province in Thailand that year (n = 9).

Over the past decade, HEC occurrences were reported in all regions of Thailand (Figure 1 and Table 1). Notably, the highest total number of occurrences was recorded in the Eastern region (n = 147), with Chanthaburi having the most HEC occurrences (n = 48) in Thailand. Conversely, the Northern region had the lowest number of cases, with only one incident reported.

HEC was also found in all periods of the year, as shown in Table 2. The highest number of occurrences was recorded between July and October (n = 162), while the period of March to June had the lowest number of incidents (n = 47).

### 3.2. Collateral Damage of HEC

Over the past decade, HEC incidents involved 9812 wild elephants and 360 humans. These conflicts have resulted in various forms of damage, including human casualties, elephant casualties, and property damage. The details of the collateral damage of HEC regarding casualties are shown in Table 3.

A total of 234 elephant casualties were recorded, comprising 166 deaths and 68 injuries (Table 3). Elephant casualties increased continuously from 2014 (n = 3) to the highest occurrence in 2022 (n = 51). Most elephant casualties resulted from electrocution (42.31%, n = 99), followed by road accidents (29.91%; n = 70), gunshots (23.50%; n = 55), and poisoning (4.27%; n = 10). Notably, incidents of electrocution, gunshots, and the poisoning of elephants have all occurred on agricultural land.

Human casualties involved 360 individuals, comprising 189 deaths and 171 injuries. Human casualties slightly increased from 23 cases in 2014 to 51 cases in 2023 (Table 3). Humans were most frequently attacked on agricultural farms (55.56%; n = 200), followed by incidents occurring in forests (27.78%; n = 100), villages (11.11%; n = 40), and roads (5.56%; n = 20).

Property damage, including broken cars and motorcycles resulting from road accidents, was reported in 14 (4.10%) HEC occurrences. Severe agricultural damage was reported in 72 instances (20.11%), despite elephants entering agricultural farms on 253 occasions (74.19%). Wild elephants intruded into agricultural farms, presumably in search of water and food, causing substantial damage to various crops, including rice, bananas, cassava, palm, durian, coconut, jackfruit, mango, rambutan, longkong, sugarcane, date palm, cashew nuts, corn, longan, pineapple, rubber trees, watermelon, cucumbers, and yardlong beans. Moreover, some wild elephants (31%, n = 106) destroyed buildings to find food; they targeted food sources such as areca nuts, unmilled rice, turmeric, and mangosteen, as well as non-plant-based items like fermented fish, shrimp paste, and fish sauce.

In 2023, the occurrence of HEC in the Eastern and Northeastern regions increased again (Table 1). The majority of occurrences in the Northeastern region (54.16%, n = 13) were linked with elephants entering agricultural land, while the Eastern region had a lower percentage of such cases (26.31%, n = 5).

### 3.3. Determining the Effect of Geography and Periods on HEC Using GEEs

There were significant geographical effects on national HEC occurrences in Thailand from 2014 to 2023 (*p* < 0.05). The Eastern region had the highest annual average occurrence of HEC (14.22 ± 1.96 per year, *p* < 0.05), whereas the Northern part had the lowest average occurrence (0.10 ± 0.10 per year) (Table 1). Pairwise comparisons indicated that the Eastern region experienced significantly more HEC occurrences than all other regions (*p* < 0.05), with the exception of the Northeastern region, where the difference was not statistically significant (Table S3).

National HEC occurrences were also statistically associated with periodical variations. On average, the highest number of HEC occurrences appeared between July and October (rainy season) (16.20 ± 2.25 per year, *p* < 0.05), while the fewest occurrences were reported between March and June (summer) (4.70 ± 1.53 per year, *p* < 0.05) (Table 2). Notably, the July-to-October period exhibited significantly higher HEC occurrences than other seasonal periods (Table S4), highlighting the pronounced influence of the rainy season on conflict frequency.

When focusing specifically on the Eastern region, the highest average numbers of HEC occurrences were observed during two main periods, November to February and July to October (Table 4), with no statistical difference (*p* ≥ 0.05). The average occurrences of HEC from November to February and from July to October were significantly greater than those from March to June (*p* < 0.05) (Table 4).

### 3.4. Determining the Relationship Between Periods, Areas, and HEC Using GLMs

In evaluating the relationship between seasonal periods and HEC occurrences, we assessed whether the geographic region (area) acted as a confounder. A comparison of models with and without area adjustment revealed that the period coefficients remained stable, with a 0% change in effect estimates. This indicates that area does not confound the relationship between the seasonal period and HEC occurrences. However, the model intercept shifted substantially (greater than 80%) upon including area, suggesting that geographic region independently influences overall HEC frequency, even though it does not alter the period-specific estimates. Accordingly, area was included in the final model to account for spatial variation, although it does not meet the formal definition of a confounder with respect to period.

The significant effects of geographic region, seasonal period, and their interactions on HEC occurrences are presented in Table S5. The model was specified using the Central region and the March-to-June period as reference categories. The intercept (estimate = 1.39, *p* < 0.05), therefore, represents the natural log of the expected number of HEC events in the Central region from March to June, corresponding to an approximately 4-fold increase in HEC events (exp(1.39)) relative to the baseline. The Eastern region, in the absence of seasonal interaction, exhibited a significantly higher occurrence of HECs compared to the Central region during the same period (estimate = 1.50, *p* < 0.05), corresponding to a 4.48-fold increase in risk (exp(1.50)) relative to the baseline.

Furthermore, significant interaction effects (*p* < 0.05) between region and the July-to-October period were observed for the Eastern, Northeastern, Southern, and Western regions (Table S5). These interactions indicate that the seasonal effect of July to October on HEC occurrences was substantially more pronounced in these regions relative to the Central region from March to June. The Western region, in particular, exhibited the strongest seasonal increase (Estimate = 3.95), translating to an approximately 52-fold (exp(3.95)) rise in HEC occurrences compared to the baseline group (Table S5).

### 3.5. Comparing the Patterns of Forest Area, Wild Elephant Population, Human Population, and Rainfall with the HEC Trend

Over the past decade, Thailand’s forest areas have exhibited fluctuations, increasing from 2017 to 2018, stabilizing for one year, and continuously decreasing until 2023 (Figure 2c). The pattern of the forest area did not match the trend of national HEC since the forest area was continuously declining, the same as the HEC occurrences. However, the pattern of national HEC corresponded to the estimated wild elephant population (Figure 2b). When national HEC peaked in 2018, the wild elephant population also reached its highest level in the previous four years (2014–2018). The population then declined around 2020 before increasing again in subsequent years, being the highest in 2023.

**Figure 3:**
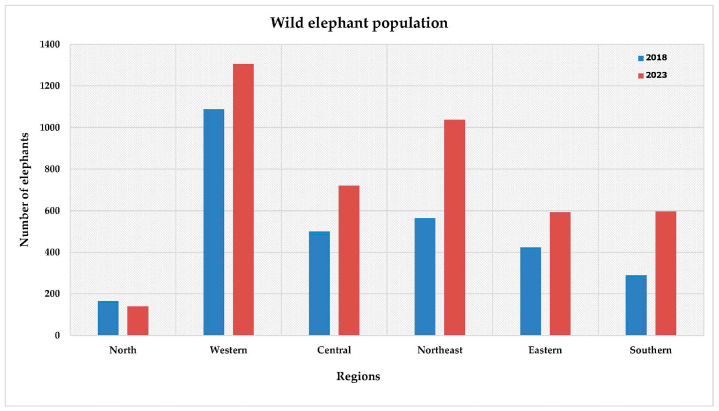
Estimated wild elephant populations in different regions of Thailand from 2018 to 2023.

Regarding the Eastern area, where the highest total HEC occurrence was reported, this region has the lowest forest area (around 7.53k km^2^) compared to the other regions, with the proportion of forest per total area being approximately 21% (Supplementary Data, Table S1). In contrast, the Northern region has the highest forest area (61,490 km^2^), with 64% of the total area covered by forests (Table S1). The highest forest area in the Northern region appears to coincide with the lowest total occurrence of HEC in that area. Additionally, the wild elephant population in the Eastern region was found to be lower than that in the Western, Central, and Northeastern regions (Figure 3).

The wild elephant population has shown an increasing trend, around 48% from 2013 to 2014, rising by approximately 5% each year (Figure 2b), particularly in the Northeastern and Southern regions, where the population nearly doubled between 2018 and 2023 (Figure 3). The highest wild elephant population was observed in the Western region in both 2018 and 2023, exceeding 1000 elephants, while the lowest population was reported in the Northern region, with fewer than 600 elephants (Figure 3). Additionally, the substantial rise in the wild elephant population in the Northeastern region in 2023 corresponded to the highest occurrence of HEC in the same area and year (Table 1).

In terms of the human population, human density has steadily increased across all regions over the past decade (Table S2), with the Central region having the highest population density (1472 persons/km^2^) and the Northern region the lowest (69 persons/km^2^). For wild elephant density, the highest density was reported in the Eastern region (0.079 elephants/km^2^), followed by the Northeast (0.042 elephants/km^2^), Western (0.041 elephants/km^2^), Central (0.037 elephants/km^2^), Southern (0.033 elephants/km^2^), and Northern regions (0.002 elephants/km^2^). Overall, the highest occurrence of HEC in the Eastern region appeared to correspond with its high wild elephant population density, while the lowest HEC occurrence in the Northern region aligned with both the lowest human and wild elephant population densities.

Regarding rainfall, the annual average rainfall from 2014 to 2023 is shown in Figure S1; the average rainfall exhibited fluctuations, with the highest levels recorded in 2017 and 2022, whereas the lowest levels were reported in 2015, 2019, and 2023. When considering the highest levels in 2017 and 2022, the highest level of rainfall happened around one year before the rise in HECs (Figure 2). When comparing each region (Figure S2), the Southern region recorded the highest average annual rainfall, followed by the Eastern region, which had the second-highest rainfall and the highest occurrence of HEC (Table 1). For each period, July to October (the highest HEC occurrence) experienced the highest rainfall, followed by March to June, while November to February was the driest.

Although the trends of the graphs, particularly for HEC occurrences and the wild elephant population, appeared similar, there was no statistically significant correlation between them (*p* ≥ 0.05).

### 3.6. HEC in the Eastern Region

The Eastern region recorded both the highest total and average number of HEC occurrences. In 2023, the region’s average monthly rainfall followed the national pattern, with the highest precipitation observed between July and October (258 mm^2^) and the lowest between November and February (25 mm^2^).

This region comprises eight provinces, many of which are adjacent to several forest areas (Figure S4). Trends in HEC occurrences for each province within the region are illustrated in Figure S5. Chanthaburi province reported the highest cumulative number of incidents, peaking in 2018. However, the number of cases declined dramatically, with no incidents reported in 2023. This province is surrounded by multiple forested areas, including Khao Chamao–Khao Wong National Park, Khao Sip Ha Chan National Park, and Khao Soi Dao Wildlife Sanctuary, which together cover approximately 32% of the province’s land area.

In 2023, the resurgence of HEC in the Eastern region was mainly driven by incidents in Prachinburi and Trat provinces. Prachinburi, which borders Khao Yai National Park, has 28% of its area covered by forest, while Trat is adjacent to Namtok Khlong Kaeo National Park, which covers 31% of its land. In terms of agricultural land, Chanthaburi has a significantly larger proportion (around 60%) compared to Trat (38%) and Prachinburi (27%). The pattern of HEC occurrences appears to be shifting between forested areas within the region.

## 4. Discussion

This study described HEC in Thailand over a decade (between 2014 to 2023). The occurrences were found in 45% of all provinces in Thailand. HEC resulted in significant collateral damage, including human and elephant casualties, as well as property destruction. Notably, national HEC was found to be significantly associated with specific areas and periods. The pattern of national HEC also appeared to be related to the wild elephant population but not to forest areas. In fact, it was more specifically linked to wild elephant population density rather than human population density.

The occurrence of HEC was statistically associated with regions, with the highest occurrence reported in the Eastern region. As most HEC incidents occurred on agricultural farms, leading to crop damage, this indicates that elephants primarily entered human settlements in search of food. This theory is supported by findings from another study, which identified 29 types of crops consumed by elephants [6]. The present study suggests that the high occurrence of HEC in the Eastern area might be associated with its status as a major crop-producing area, including rice, cassava, para rubber, sugarcane, banana, corn, pineapples, longans durian, mangosteens, rambutans, mangoes, and longkong, with peak production occurring from May to February, potentially attracting elephants in search of food [16,25]. A similar pattern has also been observed in Cambodia, where most HEC incidents occur during the crop-growing season when elephants are drawn to agricultural areas in search of food [26].

Conversely, the Northern region had the lowest HEC occurrence, possibly due to its lower wild elephant population density and a smaller quantity of fruit production compared to the Eastern region. Additionally, the types of crops cultivated in Northern Thailand may be less attractive to elephants than those grown in the East, reducing the likelihood of crop-raiding incidents [27]. A similar seasonal effect has been observed in Cambodia, where HEC follows a trend comparable to that of Thailand. This period coincides with crop growth and ripening, making agricultural fields particularly attractive to elephants [26]. Moreover, the rainy season may increase water availability, drawing elephants toward areas near human settlements where water sources are located [28,29].

In Thailand, the rainy season typically occurs from July to October, during which, all regions experience increased rainfall. This enhanced precipitation supports the growth of vegetation in both forested areas and agricultural lands, while also ensuring sufficient water availability across ecosystems. During this period, natural food and water sources for elephants should, in theory, be abundant, and consequently, incidents of HEC would be expected to decline. However, contrary to this expectation, the highest occurrence of HEC can be observed during the rainy season. This pattern may be explained by the preference of wild elephants for cultivated crops over natural forage. Numerous studies have suggested that agricultural crops are more digestible, possess higher nutritional value (particularly sugar), have fewer chemical defenses, and are more spatially concentrated and predictably available than natural plants, serving as a highly attractive and accessible food source for elephants [30,31,32,33]. This pattern is consistent with HEC incidents in India, where wild elephants frequently enter human settlements in search of food during the rainy season, particularly in agricultural areas adjacent to forest edges [10,34,35,36].

The occurrence of HEC in Thailand significantly decreased between 2019 and 2022. This decline may be attributed to the implementation of several mitigation strategies by the government, NGOs, and local communities aimed at promoting coexistence between humans and wild elephants. These strategies include crop protection methods that involve securing crops from wild elephants (firecrackers and guns to scare elephants) [24]; single-strand fencing; beehive fencing [21]; the creation of barrier vegetation; the establishment of buffer zones; and the cultivation of certain plant species as vegetative barriers, such as bamboo groves [37]. Additionally, artificial intelligence (AI), referred to as the True Smart Early Warning System (TSEWS), incorporates AI software, an internet network, and security cameras and has been integrated into systems designed to detect and notify authorities of wild elephant movements in Thailand [38]. Additionally, drones have been developed and used to manage HEC in Thailand by tracking elephant movements and managing them back to the forest [39,40,41]. To address the issue of wild elephant population control, the Thai government has conducted preliminary trials using a commercial contraceptive vaccine [42,43] and is planning its application to wild elephants in the Eastern region. However, this approach remains controversial [44]. Notably, SpayVac^®^ (ImmunoVaccine Technologies, Inc., Halifax, NS, Canada), a commercial porcine zona pellucida (pZP) vaccine, has shown promising results in African elephants, with studies indicating that a single dose can prevent pregnancy for over seven years [45,46]. This vaccine has also been used for fertility control in other wildlife species, including horses and deer [46,47].

Moreover, the decline in HEC occurrences in this study may also be associated with the COVID-19 pandemic, as strict lockdowns and travel restrictions were implemented, which initially reduced human activity in forest areas, roads, and workplaces [48]. Apart from a reduction in local people’s movements, the COVID-19 pandemic also led to a substantial decline—approximately 70%—in international tourist arrivals to Thailand, with entry to national parks being completely restricted during this period [49,50,51]. This hypothesis is supported by other studies in North America [52], Sri Lanka [53], and Thailand [51], where reduced human activity during COVID-19 lockdowns benefitted wildlife by decreasing disturbances. In Sri Lanka, for instance, the occurrence of HEC also decreased during this period [8]. However, after the end of the COVID-19 lockdown in 2023, the national occurrence of HEC rose again, with the Northeastern area experiencing the highest number of incidents. This increase is likely due to several factors, including a rise in tourist numbers and human activities; an increase in the wild elephant population; and possibly the greater cultivation of crops such as rice, maize, cassava, sugarcane, and natural rubber [54,55].

In the Eastern region, although the total number of HEC occurrences was the highest—predominantly in Chanthaburi province—the frequency of incidents showed a declining trend after 2018, possibly due to the implementation of multiple mitigation strategies in the area [7]. However, in 2023, HEC occurrences reemerged, with Prachinburi and Trat provinces showing a notable increase, despite having previously lower incident rates. These three provinces—Chanthaburi, Prachinburi, and Trat—share contiguous forested landscapes, and the observed increase in HEC incidents in Prachinburi and Trat may suggest a spatial shift in elephant movement from areas with intensive monitoring and preventive measures to those with less protection. Notably, Prachinburi is located near Khao Yai National Park, a major tourist destination that recorded a surge in visitor numbers following the COVID-19 pandemic [55]. Increased human activity and vehicular traffic in the vicinity of the park may have contributed to elevated risks of human–elephant encounters and road accidents.

One potential limitation of this study lies in its reliance on media reports as a data source for HEC incidents, which may introduce reporting bias. Media coverage often prioritizes incidents that are more dramatic or occur in more accessible or populous regions, potentially leading to an underrepresentation of human–elephant conflict events in remote or rural areas. As a result, the data may not fully capture the spatial distribution and true extent of the conflict across all affected regions but may emphasize serious incidents. This limitation highlights the need for integrating alternative data sources, such as accessible community-based reporting systems or satellite–AI monitoring systems, to provide a more comprehensive and balanced understanding of the issue. At present, fine-detailed HEC data are available from a government database; however, the dataset is limited to the past three years. Therefore, media reports remain a valuable source for conducting retrospective studies over longer time periods; this source has been used in several studies [22,56].

## 5. Conclusions

The present study indicates that HEC in Thailand is a multifactorial issue, with occurrences strongly influenced by regions and seasonal periods. Significant interaction effects between region and period suggest that these factors may synergistically amplify the risk of HEC occurrences. Additional factors such as crop production, the percentage of forest area, and solution attempts may influence HEC. The July-to-October period (the rainy season) was associated with a heightened risk of HEC, potentially due to the peak harvest season of many crops. Specifically, the Eastern region, where there is a high-density elephant population and extensive cultivation of crops attractive to elephants and which is surrounded by forest, may face a higher risk of HEC. In contrast, the Northern region, with fewer elephants, low elephant population density, and lower elephant-attractive crop production, may be at a lower risk of HEC.

Based on these findings, we propose that the July-to-October period be recognized as a high-risk season for HEC nationwide, warranting intensified monitoring and the implementation of additional preventative strategies. The Eastern region should remain a key focus area due to its persistent high-risk status and the transboundary nature of HEC across provinces. Moreover, the emergence of the Northeastern region as a new potential hotspot highlights the need for urgent attention before the situation worsens. Given the complexity of HEC and the varying influences across regions, future studies should adopt a more localized approach, focusing on both high- and low-conflict areas, to identify emerging risks and develop effective, site-specific mitigation measures.

## Figures and Tables

**Table 1:** Geographic effects on the occurrence of human–wild elephant conflict in Thailand from January 2014 to December 2023.

Year	Regions of Thailand						Total/Year
	Eastern	Northern	Northeastern	Southern	Central	Western	
**2014**	10	0	4	4	0	5	23
**2015**	13	0	1	3	2	5	24
**2016**	22	1	2	14	6	1	46
**2017**	16	0	7	5	0	13	41
**2018**	27	0	26	13	0	13	79
**2019**	10	0	9	3	1	5	28
**2020**	8	0	3	1	0	5	17
**2021**	10	0	3	2	2	2	19
**2022**	12	0	2	0	0	2	16
**2023**	19	0	24	3	0	2	48
**Total**	147	1	81	48	11	53	341
**Mean** ± **SEM**	14.22 ± 1.96 ^d^	0.10 ± 0.10 ^a^	8.10 ± 2.92 ^bcd^	4.80 ± 1.52 ^bc^	1.10 ± 0.60 ^ab^	5.30 ± 1.37 ^c^	

^a–d^ represent a statistically significant difference; *p* < 0.05.

**Table 2:** Periodical effects on the occurrence of human–wild elephant conflict in Thailand from January 2014 to December 2023.

Year	Period			Total
	March to June	July to October	November to February	
**2014**	2	15	7	24
**2015**	1	9	14	24
**2016**	10	16	20	46
**2017**	5	25	11	41
**2018**	13	29	37	79
**2019**	3	21	4	28
**2020**	1	12	4	17
**2021**	1	8	10	19
**2022**	0	9	7	16
**2023**	11	18	18	47
**Total**	47	162	132	341
**Mean** ± **SEM**	4.70 ± 1.53 ^a^	16.20 ± 2.25 ^b^	13.20 ± 3.16 ^b^	

^a,b^ represent a statistically significant difference; *p* < 0.05.

**Table 3:** Human and wild elephant casualties caused by conflict between January 2014 and December 2023.

Year	Wild Elephants			Humans		
	Injury	Death	Total Casualties	Injury	Death	Total Casualties
**2014**	1	2	3	10	13	23
**2015**	2	5	7	8	4	12
**2016**	2	5	7	7	15	22
**2017**	10	8	18	25	19	44
**2018**	4	14	18	19	19	38
**2019**	13	26	39	27	22	49
**2020**	9	27	36	11	24	35
**2021**	9	26	35	12	24	36
**2022**	8	43	51	23	27	50
**2023**	10	10	20	29	22	51
**Total**	**68**	**166**	**234**	**171**	**189**	**360**

**Table 4:** Periodical effects on occurrence of human–wild elephant conflict in Eastern part of Thailand from January 2014 to December 2023.

Year	Period			Total
	March to June	July to October	November to February	
**2014**	2	6	2	10
**2015**	1	7	5	13
**2016**	4	7	11	22
**2017**	1	9	5	15
**2018**	6	9	12	27
**2019**	2	6	1	9
**2020**	2	4	1	7
**2021**	1	1	8	10
**2022**	0	7	5	12
**2023**	2	3	14	19
**Total**	21	59	64	144
**Mean** ± **SEM**	2.10 ± 0.55 ^a^	5.90 ± 0.81 ^b^	6.40 ± 1.48 ^b^	

^a,b^ represent a statistically significant difference; *p* < 0.05.

## Data Availability

The raw data for HEC incidents are available to download at DOI:10.17632/fgcxk9f5fw.1.

## Supplementary Materials

The following supporting information can be downloaded at https://www.mdpi.com/article/10.3390/ani15091304/s1: Table S1. The forest area for each region represented as square kilometers (km^2^) or the percentage (%) of forest per total area for 2014–2023. Table S2. The human population density for each region represented as person/square kilometers (km^2^) for 2014–2023. Table S3. Significant geographic factors associated with human and wild elephant conflict (HEC) for 2014–2023 in Thailand (n = 341), using generalized estimating equations (GEEs). Table S4. Significant periodical factors associated with human and wild elephant conflict (HEC) for 2014–2023 in Thailand (n = 341), using generalized estimating equations (GEEs). Table S5. The significant relationship between the independent variables (regions and periods) and the dependent variables (HEC occurrences) and the interaction between the independent variables and the dependent variables, using generalized linear models. Figure S1. The average annual rainfall in Thailand (mm) for 2014–2023. Figure S2. The average annual rainfall in Thailand (mm), comparing among periods, between 2014 to 2023. Figure S3. The average annual rainfall in Thailand (mm), comparing regions, between 2014 and 2023. Figure S4. Map of Eastern region, including Chachoengsao, Chanthaburi, Chonburi, Prachinburi, Rayong, Sa Kaeo and Trat province. These provinces are connected to the Eastern Forest Complex (Khao Ang Rue Nai (1), Khao Sip Ha Chan (2), Khao Soi Dao Wildlife Sanctuaries (3), Khao Chamao National Park (4), Khao Khitchakut (5), Phlio Waterfall National Park (6), Khlong Krua Wai (7), and Khlong Kaew Waterfall National Park (8)), Khao Yai National Park, and Thap Lan National Park. Figure S5. HEC occurrence in the Eastern region, from 2013 to 2024.
