# Supplementary material for: Human–Elephant Conflict in Thailand over the Past Decade (2014–2023): Occurrence, Geographical Distribution, and Temporal Trends
Source: Animals (Basel). 2025 Apr 30;15(9):1304. doi: 10.3390/ani15091304 (PMC12071072; doi:10.3390/ani15091304)

Table S1. The forest area for each region represented as square kilometers (km^2) or percentage (%) of forest per total area during 2014-2023.

**Year**	**Regions**												**Whole Country**	
	**Northern**		**Northeastern**		**Eastern**		**Central**		**Western**		**Southern**			
	**km^2**	**%**	**km^2**	**%**	**km^2**	**%**	**km^2**	**%**	**km^2**	**%**	**km^2**	**%**	**km^2**	**%**
2014	62,031.07	64.57	25,198.29	15.02	7,487.75	21.72	19,043.39	20.91	32,200.97	59.12	17,695.16	23.97	163,656.64	31.62
2015	61,950.55	64.48	25,056.27	14.94	7,505.83	21.77	19,174.94	21.06	32,179.57	59.09	17,718.41	23.99	163,585.57	31.60
2016	61,840.91	64.37	25,036.52	14.93	7,531.83	21.84	19,201.48	21.09	32,146.02	59.03	17,722.93	24.00	163,479.69	31.58
2017	61,691.52	64.21	25,048.89	14.94	7,538.87	21.86	19,262.63	21.15	32,166.90	59.06	17,741.35	24.02	163,450.16	31.58
2018	61,653.49	64.17	25,200.16	15.03	7,560.26	21.93	19,462.19	21.37	32,173.62	59.08	17,931.57	24.28	163,981.28	31.68
2019	61,475.50	63.99	25,203.20	15.03	7,562.71	21.93	19,554.09	21.47	32,214.60	59.15	17,964.42	24.33	163,974.52	31.68
2020	61,331.38	63.84	25,148.88	14.99	7,559.28	21.92	19,574.11	21.50	32,196.76	59.12	17,955.16	24.31	163,765.58	31.64
2021	61,165.92	63.66	25,123.82	14.98	7,553.92	21.91	19,584.87	21.51	32,161.69	59.05	17,949.67	24.31	163,539.90	31.59
2022	61,036.26	63.53	25,113.13	14.97	7,537.97	21.86	19,637.47	21.57	32,133.56	59.00	17,959.18	24.32	163,417.56	31.57
2023	60,762.43	63.24	24,973.01	14.89	7,525.37	21.82	19,621.55	21.55	32,054.09	58.86	17,972.61	24.34	162,909.05	31.47
**Average forest area**	61,493.90	64.01	25,110.22	14.97	7,536.38	21.86	19,411.67	21.32	32,162.785	59.06	17,861.05	24.19	163,575.99	31.60
**Total land**	614,939.04		251,102.15		75,363.79		194,116.73		321,627.78		178,610.45		1,635,759.94	

Table S2. The human population density for each region represented as person/square kilometers (km^2) during 2014-2023.

Year	Regions					
	**Eastern**	**Northern**	**Northeastern**	**Southern**	**Central**	**Western**
	**person/ km^2**	**person/ km^2**	**person/ km^2**	**person/ km^2**	**person/ km^2**	**person/ km^2**
2014	140.00	65.00	120.00	125.00	1,345.97	63.00
2015	140.00	65.00	128.49	125.00	1,350.92	63.00
2016	140.00	67.40	128.53	125.00	1,354.68	82.65
2017	140.00	67.40	128.33	130.00	1,367.38	85.00
2018	140.00	69.00	127.85	132.10	1,375.20	88.50
2019	139.80	71.40	130.40	134.30	1,593.10	89.40
2020	139.90	70.90	129.40	133.90	1,585.70	88.90
2021	140.90	70.80	129.30	134.20	1,581.70	88.80
2022	141.50	70.60	129.00	134.30	1,579.80	88.70
2023	142.60	70.40	128.60	134.50	1,582.30	88.50
**Average human population density**	140.47	68.79	127.99	130.83	1,471.68	82.65

Table S3. Significant geographic factors associated with human and wild elephant conflict (HEC) during 2014-2023 in Thailand (n = 341), using generalized estimating equations (GEE).

**Geographic factors**	**Estimate**	**SE**	**P-value**
Central x Eastern	-13.600	1.947	<0.0001
Central x Northern	1.000	0.581	0.5184
Central x Northeasten	-7.000	2.832	0.1324
Central x Southern	-3.700	1.551	0.1611
Central x Western	-4.200	1.425	0.0377
Eastern x Northern	14.600	1.863	<0.0001
Eastern x Northeasten	6.600	3.339	0.3558
Eastern x Southern	9.900	2.353	0.0004
Eastern x Western	9.400	2.272	0.0005
Northern x Northeasten	-8.000	2.775	0.0454
Northern x Southern	-4.700	1.444	0.0144
Northern x Western	-5.200	1.308	0.0010
Northeasten x Southern	3.300	3.125	0.8986
Northeasten x Western	2.800	3.064	0.9432
Southern x Western	-0.500	1.943	0.9998

QIC = 1,538.50; SE = Standard error; Confidence level used: 0.95
Variables having a P value <0.05

Table S4. Significant periodical factors associated with human and wild elephant conflict (HEC) during 2014-2023 in Thailand (n = 341), using generalized estimating equations (GEE).

**Periodical factors**	**Estimate**	**SE**	**P-value**
March to June x July to October	-3.800	0.926	0.0001
March to June x November to February	-4.300	1.494	0.0112
July to October x November to February	-0.500	1.598	0.9475

QIC = 288.2; SE = Standard error; Confidence level used: 0.95
Variables having a P value <0.05

Table S5. The significant relationship between the independent variables (regions and periods) and the dependent variables (HEC occurrences), and the interaction between the independent variables and the dependent variables, using Generalized linear models.

**Factors (type of variable/number of factor levels) and significance factor level**	**Estimate**	**SE**	**t value**	**P-value**
Intercept	1.3863	0.5000	2.773	0.00556
Regions				
Central	Reference			
Eastern	1.5041	0.5528	2.721	0.00651
Northern	-24.6886	69,653.8007	0.000	0.99972
Northeasten	1.0986	0.5774	1.903	0.05706
Southern	0.6931	0.6124	1.132	0.25767
Western	-1.3863	1.1180	-1.240	0.21500

Periods				
March to June	Reference			
July to October	-1.3863	1.1180	-1.240	0.21500
November to February	0.4055	0.6455	0.628	0.52991
Interaction				
Eastern x July to October	2.3877	1.1515	2.074	0.03812
Northern x July to October	1.3863	98,505.3496	0.000	0.99999
Northeasten x July to October	2.4277	1.1674	2.080	0.03755
Southern x July to October	2.5649	1.1889	2.157	0.03097
Western x July to October	3.9512	1.5254	2.590	0.00959
Eastern x November to February	0.9383	0.6976	1.345	0.17866
Northern x November to February	22.8971	69,653.8007	0.000	0.99974
Northeasten x November to February	0.6360	0.7276	0.874	0.38207
Southern x November to February	0.9808	0.7569	1.296	0.19503
Western x November to February	2.2336	1.2199	1.831	0.06710

AIC: 103.8; SE = Standard error

Variables having a P value <0.05

Figure S1 The average annual rainfall in Thailand (mm) during 2014-2023.

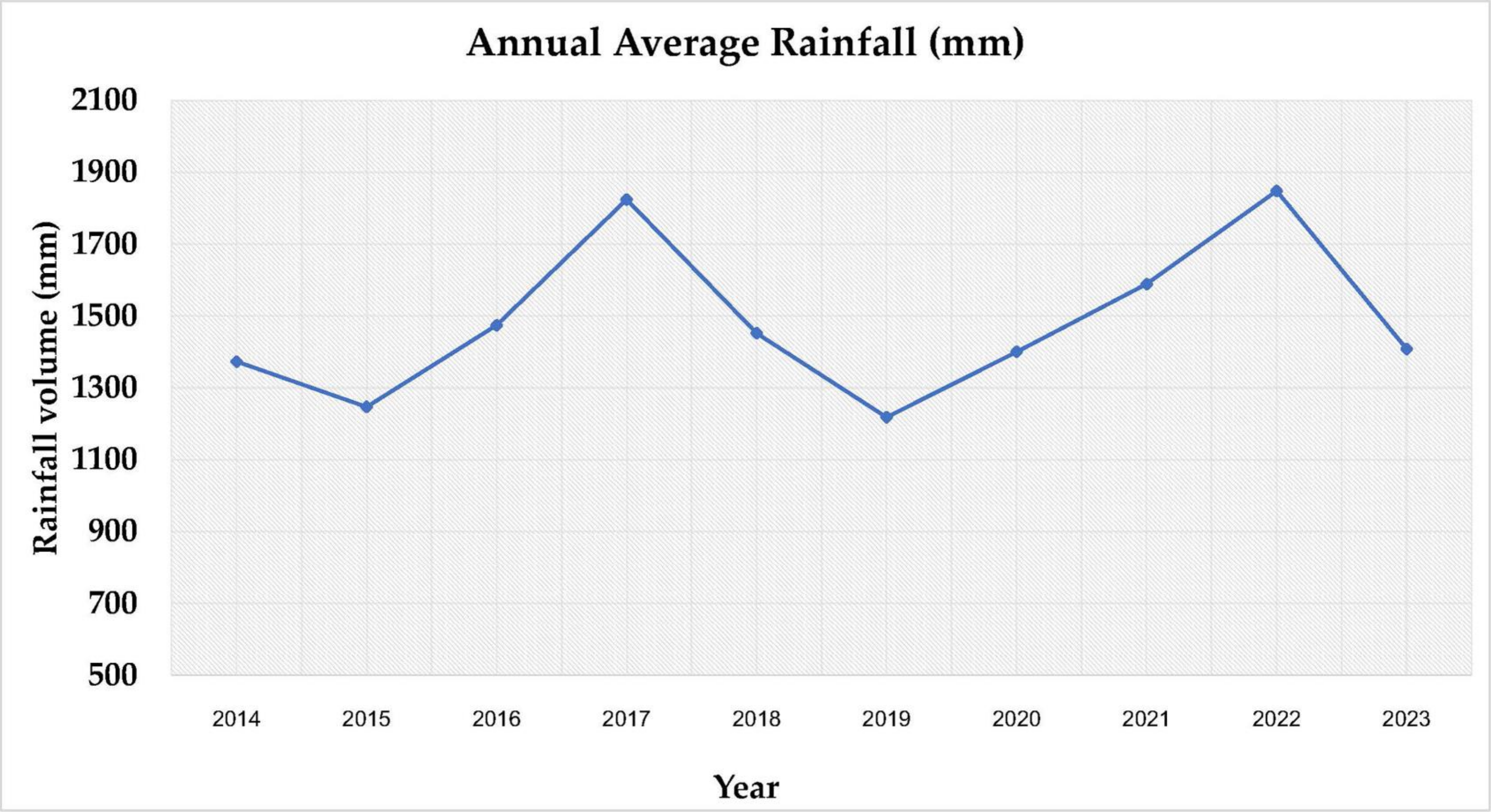

Figure S2 The average annual rainfall in Thailand (mm), comparing among periods, between 2014 to 2023.

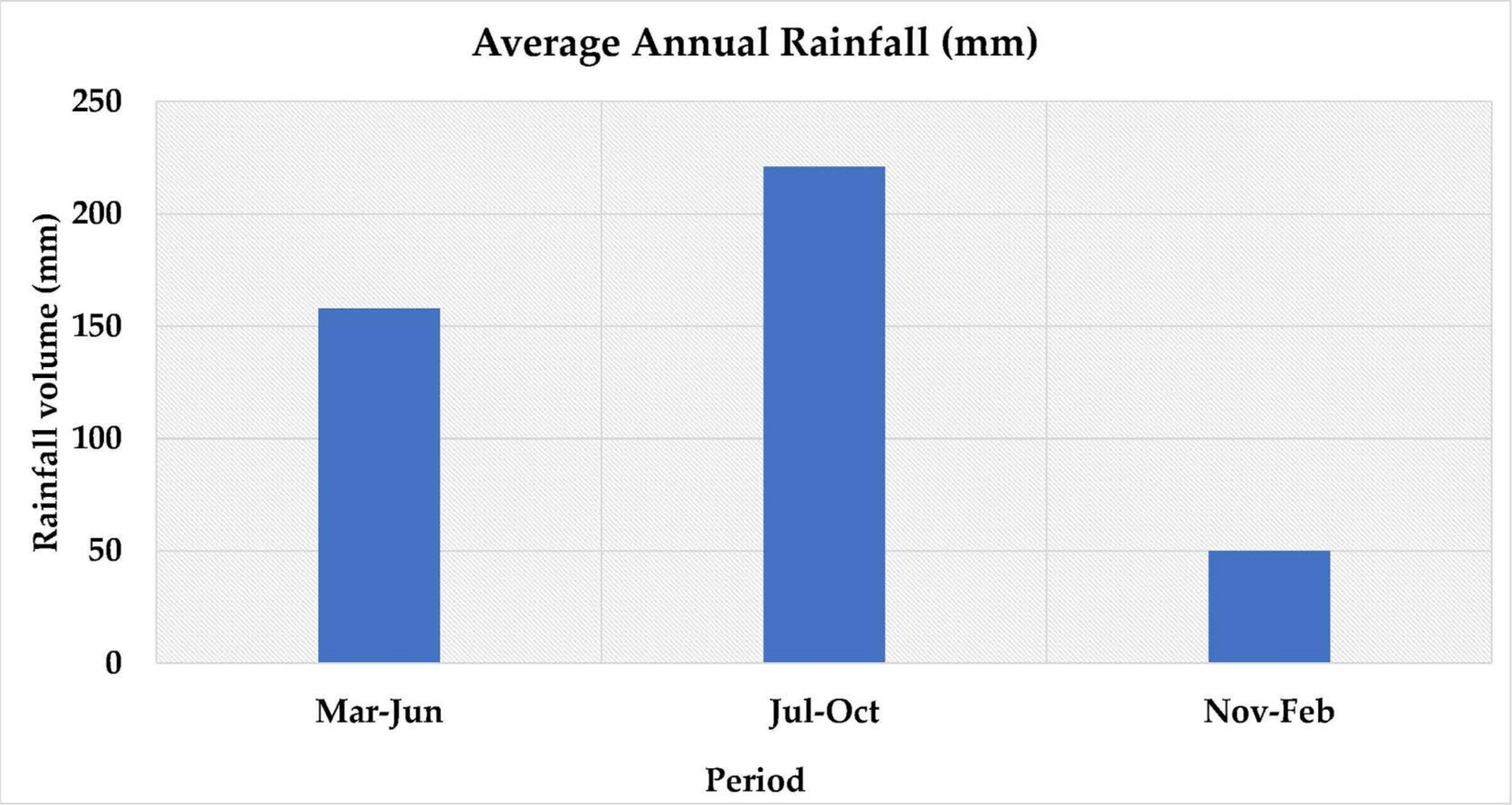

Figure S3 The average annual rainfall in Thailand (mm), comparing among regions, between 2014 to 2023.

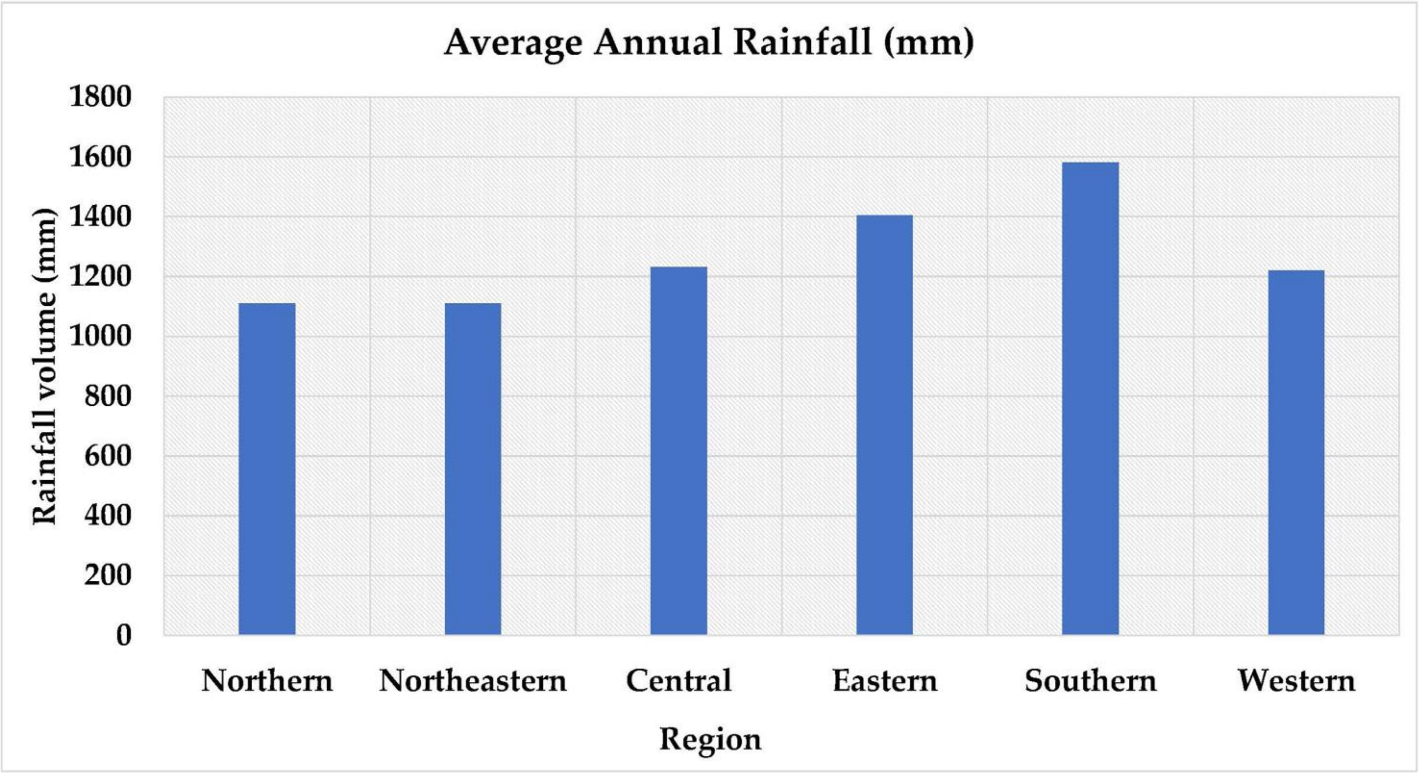

Figure S4. The map of Eastern region includes Chachoengsao, Chanthaburi, Chonburi, Prachinburi, Rayong, Sa Kaeo and Trat province. These provinces are connected to Eastern Forest Complex (Khao Ang Rue Nai (1), Khao Sip Ha Chan (2), Khao Soi Dao Wildlife Sanctuaries (3), Khao Chamao National Parks (4), Khao Khitchakut (5), Phlio Waterfall National Park (6), Khlong Krua Wai (7), and Khlong Kaew Waterfall National Park (8)), Khao Yai National Park, and Thap Lan National Park.

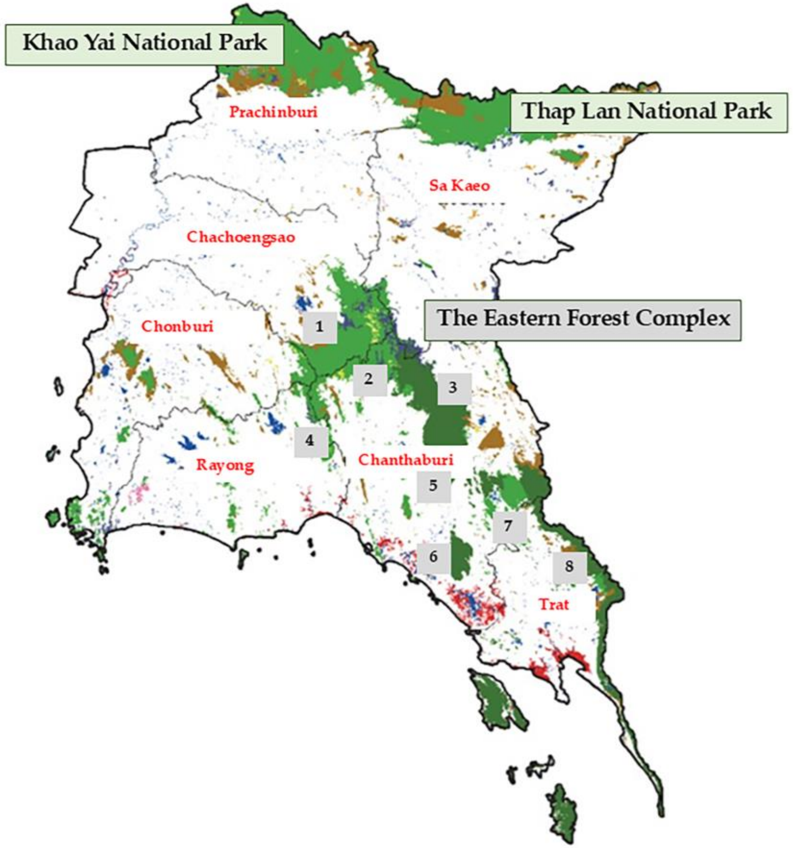

Figure S5. HEC occurrence in the Eastern region, from 2013 to 2024.

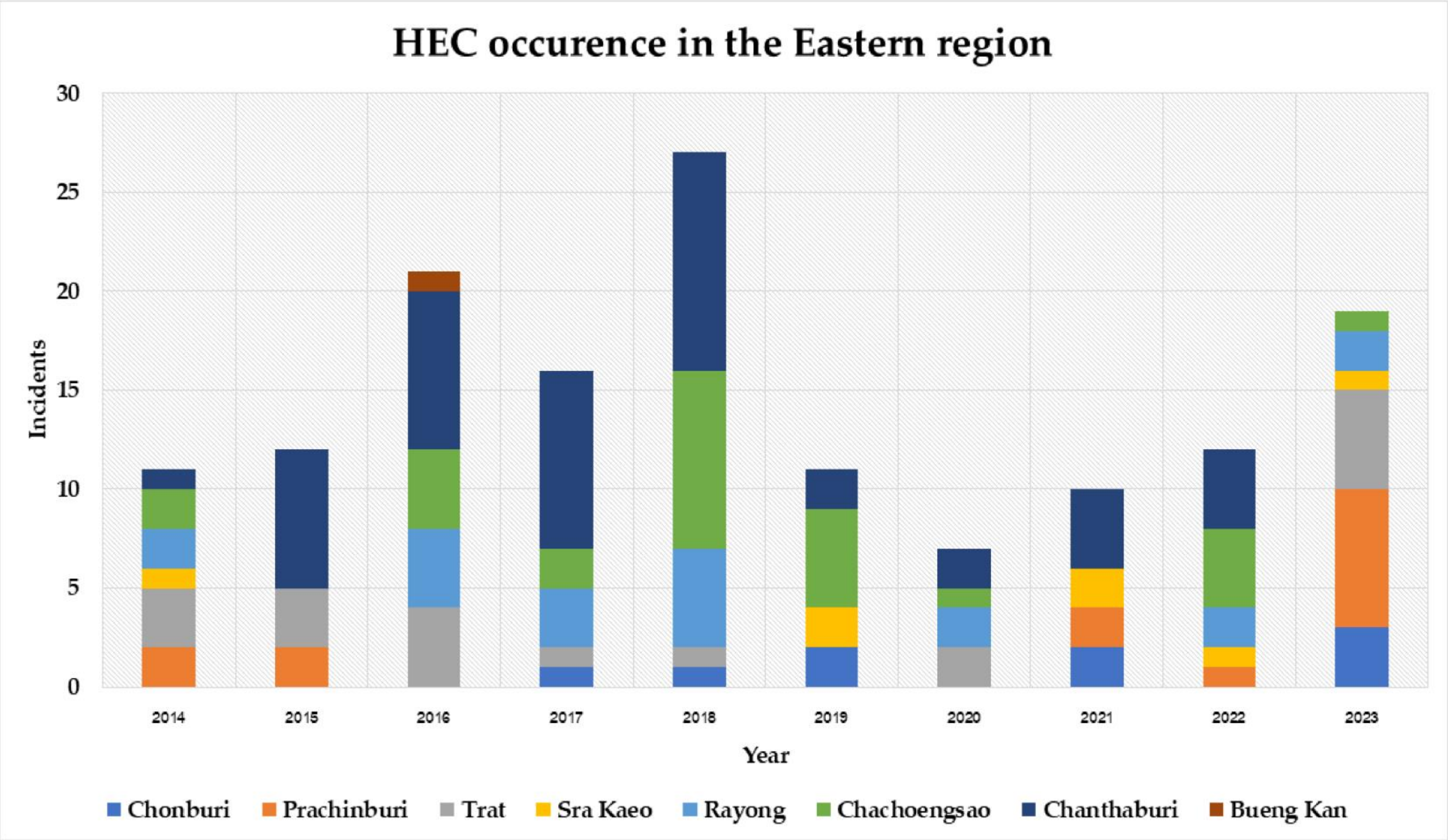

Supplement: Supplementary file 1 [file animals-15-01304-s001.zip › animals-3584924-supplementary.pdf]
